# Using functional near-infrared spectroscopy to study the early developing brain: future directions and new challenges

**DOI:** 10.1117/1.NPh.10.2.023519

**Published:** 2023-04-03

**Authors:** Judit Gervain, Yasuyo Minagawa, Lauren Emberson, Sarah Lloyd-Fox

**Affiliations:** a University of Padua, Department of Developmental and Social Psychology, Padua, Italy; b University of Padua, Padova Neuroscience Center, Padua, Italy; c Université Paris Cité, CNRS, Integrative Neuroscience and Cognition Center, Paris, France; d Keio University, Department of Psychology, Faculty of Letters, Yokohama, Japan; e University of British Columbia, Department of Psychology, Vancouver, British Columbia, Canada; f University of Cambridge, Department of Psychology, Cambridge, United Kingdom

**Keywords:** functional near-infrared spectroscopy, developmental neuroscience, infants, children, brain imaging

## Abstract

**Significance:**

Functional near-infrared spectroscopy (fNIRS) is a frequently used neuroimaging tool to explore the developing brain, particularly in infancy, with studies spanning from birth to toddlerhood (0 to 2 years). We provide an overview of the challenges and opportunities that the developmental fNIRS field faces, after almost 25 years of research.

**Aim:**

We discuss the most recent advances in fNIRS brain imaging with infants and outlines the trends and perspectives that will likely influence progress in the field in the near future.

**Approach:**

We discuss recent progress and future challenges in various areas and applications of developmental fNIRS from methodological and technological innovations to data processing and statistical approaches.

**Results and Conclusions:**

The major trends identified include uses of fNIRS “in the wild,” such as global health contexts, home and community testing, and hyperscanning; advances in hardware, such as wearable technology; assessment of individual variation and developmental trajectories particularly while embedded in studies examining other environmental, health, and context specific factors and longitudinal designs; statistical advances including resting-state network and connectivity, machine learning and reproducibility, and collaborative studies. Standardization and larger studies have been, and will likely continue to be, a major goal in the field, and new data analysis techniques, statistical methods, and collaborative cross-site projects are emerging.

## 1 Introduction

Since its first application to study infant development (0 to 2 years),^1^ functional near-infrared spectroscopy (fNIRS) has rapidly become a highly popular method for developmental neuroimaging. It is used particularly frequently to image the brain of very young individuals, as the relatively thin skull and tissues of infants and toddlers allow light to penetrate deeper into the cerebral cortex than in older populations. Furthermore, other imaging methods, such as functional magnetic resonance imaging (fMRI), are often too constraining or invasive to be used with infants and young children. fNIRS is also easy to use and relatively tolerant to motion. It is thus a particularly suitable methodological choice for developmental populations.

**Fig. 1:**
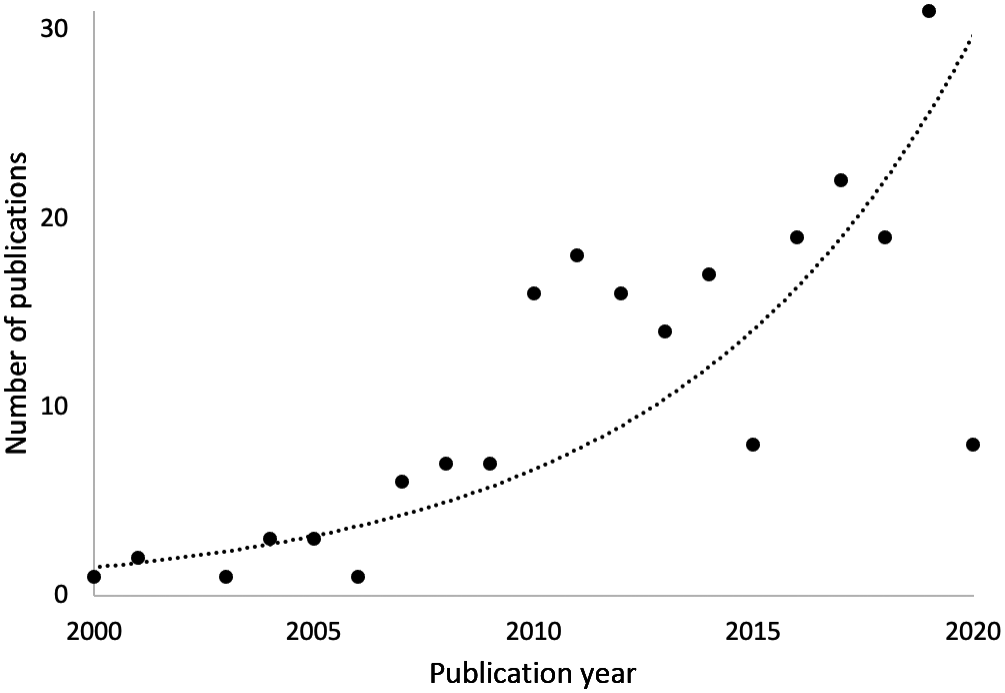
Increase in number of published infant studies (with 0- to 2-year-old) using fNIRS. Since its inception as an infant neuroimaging technique, fNIRS has been used in an increasing number of infant cognitive neuroscience experiments. Figure adapted with permission from Ref. 5.

These advantages notwithstanding, infants, and toddlers are challenging research participants who have a short attention span, do not understand and/or necessarily comply with instructions, do not easily stay still or may be unwilling to accept the fNIRS cap. In the two and a half decades, since its first use with infants, fNIRS technology, experimental methods, and data analysis techniques have evolved considerably^2^^–^^4^ to meet the special needs and challenges of developmental neuroimaging. As a result, developmental fNIRS research is growing exponentially^5^ (Fig. 1). Indeed, since 2010, growth has been particularly fast, possibly related to increasing fNIRS expertise in the developmental community as well as the appearance of a greater variety of commercially available systems in more accessible price ranges. Relative to fMRI and electroencephalography (EEG), since 2017, the distribution of new infant development publications (with 0- to 2-year-old) showed a higher increase in fNIRS suggesting a potential shift in the choice of methods for infant studies.^6^

In this paper, we consider some of the most recent advances in developmental fNIRS and outline the challenges and perspectives that developmental fNIRS research is likely to face in the near future. Our aim is not to provide a review of the existing developmental fNIRS literature, but rather to highlight current trends and challenges that we believe will shape the future of the developmental fNIRS field.

### 1.1 Previous Perspectives on Developmental fNIRS

Although fNIRS had been applied to developmental research soon after its first appearance, early studies were mainly confirmatory, seeking convergence with existing empirical results established through other imaging and behavioral methods and establishing regional selectivity/localization for certain cognitive or perceptual functions (for reviews, see Refs. 2, 3, 7, and 8). Indeed, a 2012 review paper^4^ states that “…[t]he most important question one can ask about [f]NIRS findings from infants is: What have we learned that we did not know already from other measures? That is, what is the “value-added” of [f]NIRS for studies of infant perception, cognition, and language?.” This is a key question and one that certainly has not left the field. A number of the new directions emerging in the field of developmental fNIRS are motivated by exactly these questions. Here we will consider a number of exciting new directions through this lens: how are they value-added? We take value-added to mean studies that reveal more about the topic of interest (e.g., a clinical population, underlying cognitive mechanisms, and developmental changes) than what could be gleaned without using fNIRS. The previous review^4^ also raises the issue of replicability, and how the developmental fNIRS field can move toward more hypothesis-driven research rather than exploratory. A decade ago, another challenge facing the field was conducting studies across multiple age groups cross-sectionally and, whenever possible, longitudinally. Beyond the usual practical and methodological difficulties of conducting studies with multiple age groups, this raises a particular challenge for fNIRS. For example, there are developmental changes in neurovascular coupling, an issue that remains poorly understood even today,^9^^,^^10^ and changes in the neuroanatomy of the brain which make localization across ages a challenge.^11^^,^^12^

The increasing technological advances of NIRS technology and, neuroimaging in general, have also brought about new challenges and opportunities. There is an ever-stronger impetus for research with human participants to be inclusive, investigating the perceptual, cognitive, affective, and neural abilities of individuals who rarely participated in laboratory studies before. This has led to developmental fNIRS research being taken outside the laboratory to reach global populations, to test participants in their natural environments, and to assess individual variation.

These advances also require increasingly complex and sophisticated methodological procedures and data analysis techniques, many of which are being adapted to fNIRS from other imaging modalities and computational approaches, such as resting state and functional connectivity analysis or machine learning.

The field is currently making considerable efforts to address the issues outlined above, as overviewed in the following sections.

### 1.2 Current Paper

We review the most recent challenges and opportunities in developmental fNIRS with the aim of providing insights into the trends that will likely shape the future of the field. Specifically, we present the technological advances, for instance, hyperscanning and wearable systems, and the applications that have been brought about by the growing need to conduct more inclusive and more ecologically valid research, taking fNIRS out of laboratories, “into the wild” and across the globe. We discuss the increasing attention paid to individual variation and developmental trajectories, including longitudinal research spanning several time points, sometimes across a number of years. We also review the data processing and analysis techniques that have accompanied these advances as well as the latest initiatives aiming to guarantee the reliability and reproducibility of fNIRS results.

## 2 Bringing Developmental Neuroimaging into More Ecologically Valid, Diverse, and Naturalistic Contexts

Although research within a laboratory setting has provided great advances in our understanding of human development, extending the ecological validity of these results is of increasing importance. Research in laboratories is well-suited to some developmental phenomena. Indeed, many of young infants’ earliest abilities have been revealed in controlled research environments (i.e., video-screen-based stimuli with strict parameters to account for perceptual auditory and visual differences), using NIRS (for reviews, see Refs. 2, 3, 7, and 8), and this will continue to be an appropriate design for many theoretically motivated questions even in the future. Several of these lab-based studies have taken advantage of the flexibility of fNIRS to investigate questions using more realistic, ecologically valid stimuli (e.g., puppet theater to test object perception,^13^ live presentation of goal directed actions,^14^ and live interactions between researcher and infants to measure ostensive signals^15^). Indeed, one of the earliest fNIRS studies with infants combined a naturalistic, live researcher–infant interaction paradigm (A-not-B task) with a longitudinal design to investigate the neural underpinnings of this behavioral phenomenon in infancy.^16^

However, other phenomena, e.g., those that are inherently multimodal, multidimensional, or dynamic, may only be partially explored in the restricted context of a laboratory, impeding our full understanding of the research questions posed. For instance, in social cognition, a central theme in developmental psychology and an area of exponential growth in infant fNIRS,^5^ infants’ social skills have classically been assessed using static pictures or video stimuli of social signals to better control the experimental contrasts under investigation. However, research has shown the critical role of live social stimuli in infants,^17^ as exemplified by the phenomenon “video deficit,” whereby infants tend to show difficulties in learning or performing via unidirectional, nonlive video stimuli/presentations. For example, live “peek-a-boo” elicited more prefrontal responses in infants than nonlive presentations^18^^,^^19^ and live action observations produced adult-like activation patterns in motor areas while a televised version did not.^14^ These differences can be explained by factors, such as perceptual saliency (e.g., 2D versus 3D), bidirectionality, and contingent responsiveness,^20^ and highlight the importance of embedding ecologically valid designs in our research program to better study context-sensitive, multidimensional, or dynamic phenomena.

Research in labs can also result in highly biased sociocultural sampling. For example, research labs are predominantly located in universities or hospitals in cities and therefore recruit urban and suburban populations. Furthermore, socially, culturally, or financially disadvantaged groups—those most at risk for experiencing socioeconomic and health challenges—are far less likely to participate in research.^6^^,^^21^ To include previously underrepresented ethnic-racial and geographical groups in developmental research,^22^ we must seek to reach out beyond lab-based settings.

In recent years, the portability of fNIRS has allowed researchers to use more naturalistic contexts and to bring neuroimaging technologies to populations not historically included in developmental research.^23^ For example, fNIRS was recently applied in a rural context in Côte d’Ivoire, where the instrumentation was transported village to village in a jeep.^24^ Moreover, fNIRS technology and technological innovation is more commonly taking place in lower-resource research labs that formerly did not have access to such technology, such as the use of high-density optical imaging in a cohort of 7- to 10-year-old children in Colombia.^25^

Below, we highlight two particular technological implementations—wearable fNIRS and hyperscanning—which have led to a recent surge in more ecologically valid, inclusive, and diverse research.

### 2.1 Global fNIRS

“Global fNIRS” has become a catch-all term to describe the use of fNIRS in contexts that are underrepresented in the field of developmental neuroscience and cognition, in particular within low- and middle-income countries, low financial/health/education resource settings, and/or rural/hard-to-reach communities (for a recent review of the inclusivity of developmental neuroimaging studies, see Ref. 6). The majority of research on children’s cognitive development conducted in global health contexts had been limited to behavioral assessments to measure the effect of exposure to early adversity or strengths-based investigations of cognition. Such measures are often undertaken later in childhood rather than at the time that vulnerability to exposure is most critical (i.e., during prenatal and early postnatal life^26^). Beginning with a study published in 2014, by researchers working in The Gambia to understand the deleterious impact of undernutrition on brain development,^27^ fNIRS has begun to be increasingly used in “global” contexts. The majority of these studies are embedded within larger-scale global health studies, which has meant that the technique is also increasingly applied in longitudinal (0 to 5/7 years) and/or multiple-age-group (5 to 15 time points) experimental designs.^27^^–^^32^ Further, in a new clinical efficacy trial of nutritional intervention in The Gambia (Improving Infant Neurocognitive Development and Growth Outcomes with Micronutrients Trial; 10.1186/ISRCTN15063705), fNIRS will be applied longitudinally in a sample of 600 infants, which, to our knowledge, will be the largest fNIRS developmental study to date. By integrating fNIRS into these multimeasure, large-scale studies, researchers hope to better understand early trajectories of brain development. The aims are to both understand the deleterious impacts of adversity, such as psychosocial and environmental risk, and the potential beneficial impact of factors, such as enriched resource family context (i.e., multiple caregivers and supportive community practices). To better represent our understanding of child development by diversifying populations under study,^33^ we must create better pathways for access to neuroimaging for Majority World countries (those outside of G8 countries, where 85% of the world’s population live but currently only 5% to 15% of child developmental research has been conducted^33^^–^^35^), including reducing the cost and accessibility of technology that to date has generally been developed and sold in Minority World countries (such as Europe, United States Canada, Australia, and Japan).

### 2.2 Advances in Wearable fNIRS Technology

**Fig. 2:**
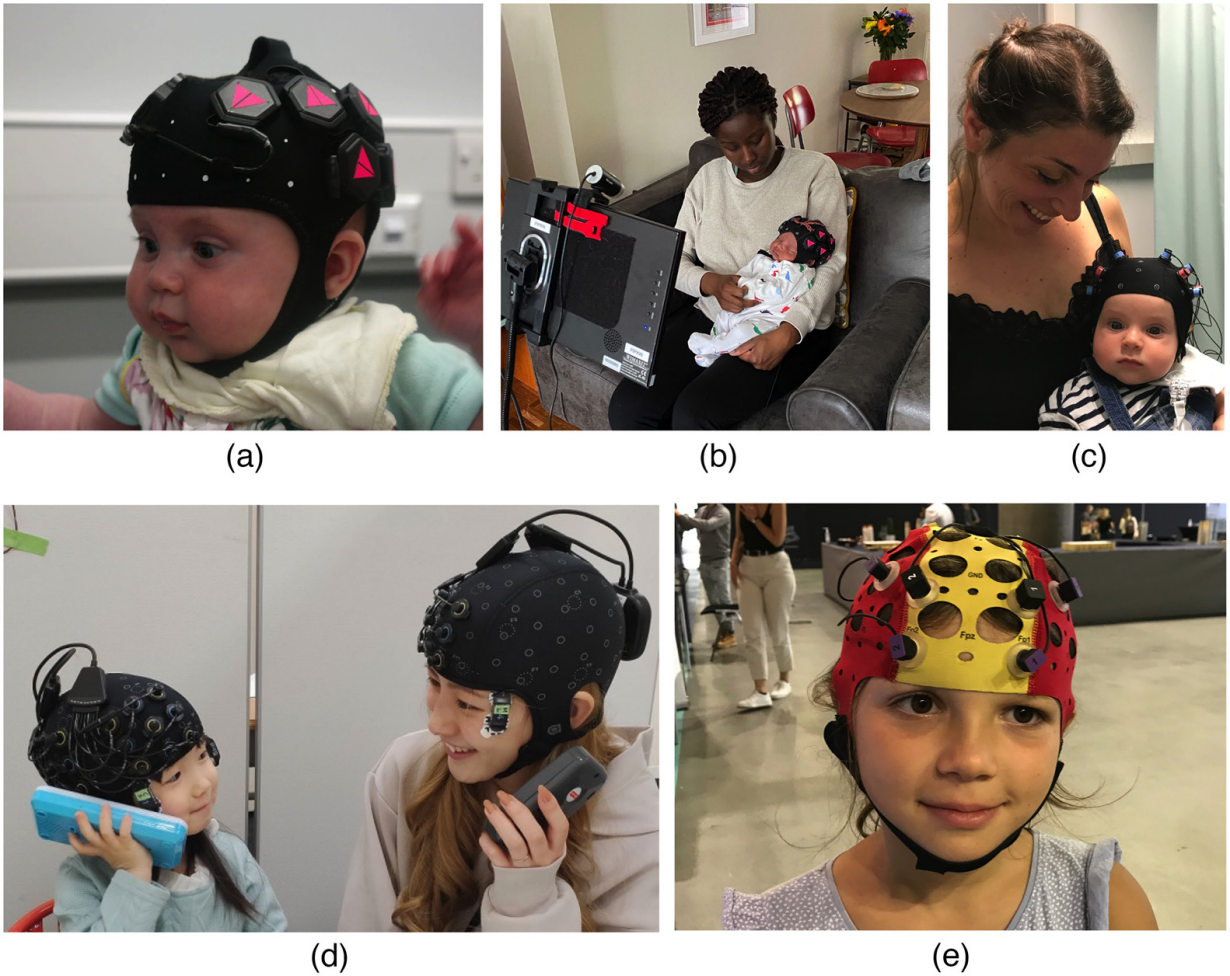
Families taking part in studies using wearable fNIRS systems: (a) at a research lab visit (LUMO, Gowerlabs) and (b) during a home visit as part of the longitudinal PIPKIN Study,^36^ (c) in a lab study on language development (NIRSport 2, NIRx), while (d) hyperscanning mother and child (Brite MKII, Artinis) combined with a wireless EMG system (pico EMG, Cometa; at the outer corner of the eyes) to measure facial muscle activity, and (e) while freely locomoting inside a building (PHOTON, Cortivision).

Wearable or mobile fNIRS refers to miniaturized wireless devices that are embedded within the headgear or other apparel that the participant wears (Fig. 2) and has been widely used in adults, and increasingly used in developmental populations.^37^^,^^38^ This is in contrast to more conventional table-top/trolley fNIRS devices that are relatively heavy and large in size. Moving toward, more wearable devices have numerous scientific benefits. First, wearable devices further increase the robustness of fNIRS to participant motion facilitating investigations of naturalistic activities, such as walking and crawling. Wearable neuroimaging devices may also be well placed for answering educational questions in nursery and school settings.^39^ Relatedly, wearable fNIRS will likely allow longer periods of measurement, which is particularly important in clinical settings, such as the monitoring of premature infants within neonatal intensive care. As development is inherently dynamic, documenting developmental change as it unfolds, e.g., learning, recovery from trauma, or improvement due to rehabilitation, rather than detecting its effects *a posteriori* will allow us to better understand the mechanisms at stake, and leverage them for more efficient interventions. Wearable fNIRS will also allow hard-to-reach developmental populations to be better studied, such as individuals with mental health challenges (see Sec. 3.1), individuals who may consent to partake in community-based research but are not comfortable attending more formal institutes and those living in lower income and Majority World countries (see Sec. 2.1). Finally, due to the miniaturization of hardware, concurrent recording of other physiological measures, such as electrocardiography, electromyography (EMG), and EEG is now far more achievable (Fig. 2).

Although the movement to wearables already has a number of scientific benefits, technological improvement is still ongoing, e.g., by increasing crucial factors, such as the sampling and coverage of the cortex. For example, the technological advancement of diffuse optical tomography within wearable hardware has increased signal-to-noise ratios, coupled with reduced exclusion rates in human infants.^40^ Thus the cutting edge of fNIRS systems in terms of the density and quality of recordings are emerging together with the move toward wearable, infant-friendly fNIRS systems.

### 2.3 Hyperscanning

Similarly supported by the relative physical ease of recording, fNIRS neuroimaging during a live social interaction or hyperscanning has been growing rapidly in the last decade and a literature focusing on hyperscanning of infants and caregivers is emerging [Fig. 2(d)]. Hyperscanning studies with adults have provided significant knowledge in social neuroscience.^20^ Although there are currently a small number of studies on fNIRS hyperscanning between older children or between an adult and an older child,^41^^–^^44^ those with infants are more limited.^45^ For example, a preliminary study^20^ measuring mother–infant synchronization while mothers were holding their 2- to 3-month-old infants indicated stronger interpersonal synchronization of the anterior left OFC during the holding condition (versus the separation condition). Similarly, most hyperscanning studies with young populations have reported higher interpersonal coherence during cooperation or interactive behavior. These studies established a fundamental initial step in this method. However, some crucial issues require further efforts. First, precise behavioral coding of social signals (e.g., eye contact) including microsignals, preferably employing AI-based automatic coding, should be associated with fNIRS data to better establish mechanisms of synchronization.^46^ To fully understand the signals obtained, analysis methods, such as transfer entropy and mutual information, assessing causal relationships and amount of information exchange between data from multiple participants needs to be better established. Currently, published data most often center on global patterns of synchronization across all channels. The full potential of the spatial resolution of fNIRS should be utilized to investigate region-specific synchronization to identify more precise brain networks at play, and how these change over development. Finally, as a common approach for hyperscanning designs is to use them in more interactive “in the wild” studies, standardized methods for denoising and the removal of motion or systemic artifacts should be established, as natural interaction is accompanied with a dense and divers range of body movements and systemic signals. Predefining the frequency range for measures of synchronization, such as wavelet transformed coherence, will contribute to solving this issue.

## 3 Individual Variation: Uncovering Different Developmental Trajectories

Most traditional developmental studies investigate phenomena at the level of a group of participants. There is, however, increasing interest in individual variation and the diversity of developmental trajectories, coupled with a need to understand why these differences exist. For developmental fNIRS research to better assess individual variation, we need to elucidate differences in the aetiology of vascular and metabolic pathways within specific and more general populations. At least two new directions have addressed these challenges: large-scale longitudinal studies (such as some global fNIRS studies) and research focusing on individuals with a broad range of developmental trajectories (e.g., infants born prematurely, children with developmental disorders, difficulties with hearing, or vision, etc.; for review see Ref. 47).

### 3.1 Developmental Disorders

Some children with developmental disorders may find an fNIRS study easier to participate in than an fMRI study due to challenges associated with their condition [e.g., co-occurrence of anxiety or aversion to loud noises and confined spaces for some individuals with autism spectrum disorder (ASD/ADHD)]. Indeed, fNIRS has been successfully used in several developmental disorders, identifying altered brain function, anatomy, or connectivity in individuals with developmental disorders, e.g., in autism spectrum disorder,^48^^,^^49^ including nonverbal populations,^50^ to study social cognition, language, and executive function. Furthermore, several studies^51^^–^^54^ have reported different patterns of cortical responses to social signals in infants at elevated familial likelihood of developing ASD around 5 months of age, relative to typical likelihood infants. Importantly, in one study, infant responses early in development correlated with traits of ASD at 3 years of age.^53^ In addition to evidencing the development of the social network in ASD, these studies also indicated the feasibility of the fNIRS signal as a neural marker to detect ASD. These findings have highlighted a potential strength for the use of fNIRS in populations where different patterns of brain development may be evident at an earlier age than behavioral changes, and thus could help target family support and interventions for those infants and families in need.

### 3.2 Assessing Clinical Interventions

fNIRS has also been applied to study individual variation in clinical interventions or treatment. It is particularly well suited to image infants with cochlear implants, as other imaging modalities are not compatible with the device (fMRI due to the metallic parts and EEG due to signal interference). In infants with severe hearing impairment who experience auditory deprivation and the subsequent improvement of hearing following cochlear implantation, fNIRS has great potential to improve therapeutic outcomes and further our understanding of language development.^55^^,^^56^ Indeed, one study observed that the amplitude of the fNIRS response to human voice versus nonvoice stimuli in hard-of-hearing infants at 12 months was correlated with their residual hearing.^55^

### 3.3 Preterm Birth and Perinatal Transitions

A further clinical area of interest for studying individual variation is preterm birth as this has been associated with risks for cognitive impairments, including language delay and ADHD. In one such study,^57^ infants born prematurely (<33 weeks gestation) were tested in an audiovisual learning and prediction task. The authors found that while the neural and behavioral signatures of audiovisual learning were intact, infants did not show neural signatures of sensory prediction (also see Refs. 58 and 59).

The study of individual variation, in particular within premature infants, highlights an opportunity for study but also a challenge for fNIRS research. In particular, the metabolic and synaptic systems of the brain are rapidly developing during the prenatal and early postnatal periods. Perinatal fNIRS of preterm and term neonates has provided insights into cortical angiogenesis and neurogenesis. A series of studies with this population highlighted that neural responses measured by EEG and hemodynamic responses measured by NIRS can be decoupled in premature newborns.^60^^–^^62^ Under some conditions, close-to-typical EEG responses were associated with weak NIRS responses and reduced blood flow, suggesting that neurovascular coupling is immature and highlighting the risk that when active, neurons may remain without the necessary metabolic nutrients in premature neonates. Furthermore, the Hb phase of oxygenation and deoxygenation, referred to as hPod,^63^ showed changes from an in-phase to an antiphase pattern according to postnatal age (PNA), but early preterm infants showed slower changes. Although this Hb phase development gradually continues at least until 10 months of age,^64^^,^^65^ its relationship with cognitive function remained unclear. Examining resting-state connectivity in preterm and term neonates, one study^66^ suggested that how perinatal neurovascular and metabolic systems relate to neurocognitive networks may be captured by the long-range connectivity.

In contrast to the canonical hemodynamic response, characterized by an increase in oxy-Hb with a slight decrease in deoxy-Hb, younger infants tend to show variable, or sometimes opposing patterns of activation, which can be mediated by gestational and PNA, stimulus quality, or task difficulty.^67^^–^^69^ Vascular changes over development pose a particular challenge for longitudinal work and for drawing comparisons across populations/ages.

To assess individual variation in more depth, future work should place more focus on larger cross-sectional samples and/or longitudinal frameworks to track age-related changes, taking contemporaneous measurements of brain function and behavior in parallel with measurements of the environment, such as adversity, health, community, and family context to further understand why differences exist.

## 4 Statistical and Analytic Advances

Although the previous sections have noted a number of technical, methodological, and analytic challenges, the field of infant fNIRS (and fNIRS as a whole) has been making substantial progress on these fronts. Here we outline a selection of recent statistical and analytic advances that promise to further the field.

### 4.1 Resting-State Networks

The resting-state network (RSN) is the measurement of networks in the absence of sensory or cognitive constraints, tasks, or stimulation. These networks are principally based on structurally connected regions, reflect coordinated functional networks, such as default-mode networks and executive control networks, and have widely been studied with fNIRS (as well as fMRI). To summarize the current findings, there appears to be a shift from stronger short-range to long-range connectivity across infancy but some connections show U-shaped development.^66^^,^^70^^–^^72^ Importantly, state of alertness (sleep/wakefulness) also affects the RSN.^71^^,^^73^

Several analysis methods have been proposed and utilized in fNIRS, including a general linear model (GLM)-based analysis, dynamic-causal modeling,^74^ independent component analysis,^75^ joint probability distribution of phase,^76^ and dynamic functional connectivity^77^ to establish resting state connectivity. Directionality of the connectivity can be assessed using methods, such as directional phase transfer entropy and directional connectivity. In infant fNIRS studies, a popular method is GLM-based analysis mostly using the cross-correlation of Hb changes. The phase-based analysis using instantaneous phase is also a useful method for assessing functional connectivity from within averaged block design data as well as long-term measurement of more standard data of longer duration.^69^

One challenging issue with infant RSN is measurement during awake states. Awake RSN without any stimulus is feasible with very young infants (e.g., neonates), but as infants age, they require some form of stimulation to prevent them from becoming inattentive and restless. Visual stimulation that catches infants’ attention has been used^72^ but has limitations with regards to a full understanding of what is being captured relative to true RSN paradigms. Overall, a standard set of approaches would be beneficial to allow across-lab data collection.

Unlike fMRI, fNIRS data benefits from the measurement of both oxy-Hb and deoxy-Hb, and therefore, could provide insights into RSNs, yet to-date some studies report only oxy-Hb. NIRS even allows for additional measures to be used in identifying RSN, e.g., total-Hb^78^ and cytochrome-C-oxidase (oxCCO).^54^ It will be highly important to understand how we can effectively utilize all four measures of hemoglobin and oxCCO for connectivity analysis, including frequency-dependent connectivity.

### 4.2 Task-Based Functional Connectivity

Complementing the resting-state connectivity approaches, there are emerging analytic techniques for understanding neural networks that infants employ while they are engaged cognitively. Here we refer to this technique as task-based functional connectivity. For example, a recent study^79^ found a large frontoparietal network in young infants that was engaged when infants were participating in a sequence prediction task (versus perceptual control task). Importantly, this differential functional engagement was found within a single experimental session showing dynamic engagement according to an infant’s cognitive state. This technique holds promise to expand our understanding of the neural mechanisms supporting infant cognition and how they change with development from investigating activity of individual regions to how regions collectively engage and form large-scale networks. However, the relevant analytic procedures are still being developed in infant fNIRS with many decision points needed to be mapped out (see Ref. 80 for a systematic investigation of some of these decision points). How functional networks change in time, not only throughout development, but also dynamically during a task is of great relevance. This could be investigated on a trial-by-trial basis or for a longer period of time (e.g., over 5 to 8 consecutive blocks^79^), over an uninterrupted timecourse.

### 4.3 Machine Learning and Decoding Approaches

Machine learning and decoding approaches have a number of benefits including allowing researchers to look at patterns of activity across channels and using data-driven versus hypothesis-testing approaches. In general, a subset of the data (either a subset for each infant or a subset of the infants in the sample) is used to create or train a computer model and then this model is used to analyze or “decode” the remaining data. A recent study^81^ found evidence that infant data can be readily decoded using simple machine learning techniques and presented a task where univariate analyses (i.e., each channel analyzed separately) would not have revealed differential neural activation while decoding approaches did. As the types of machine learning techniques applied to neural data grows, so too do the possibilities for this technique. For example, explainable artificial intelligence models were used^82^ to reveal new relationships between patterns of activation in a previously analyzed dataset. Although there is a lot of promise in this approach, there are a number of challenges. First, many machine learning approaches need a large amount of data to train elaborate computer models, which is challenging for infancy researchers. Also the statistical tests are not as clear-cut with these techniques, and traditional power analyses are not applicable (see Ref. 81 for bootstrapped statistical tests). Finally, it is important to think clearly about what scientific questions these techniques can answer and how they are similar or different from more traditional-hypothesis testing techniques. With a growing complement of analytic approaches, the linking hypothesis between question and test is ever more important.

### 4.4 Reproducibility, Meta-Analyses, and Collaborative Replication Projects

Concerns about reproducibility have recently emerged in many areas of empirical research from psychology and sociology to neuroscience and medicine, as some key findings were found not to replicate reliably.^83^^–^^86^ A number of the factors that have led to the replicability crisis are particularly serious in developmental research, as young children are challenging participants, which may lead to large amounts of missing data, imprecise data rejection criteria, short study durations, small numbers of test trials, small sample sizes, and relatedly, low statistical power. Developmental fNIRS research is no exception. There is thus a clear need in the developmental fNIRS community to address issues of replicability. A number of complementary solutions are emerging to address this issue.

One approach is to conduct a meta-analysis of existing published and/or unpublished studies that address the same research question. A meta-analysis is a quantitative method for gathering data from experimental studies into a larger dataset in order to calculate the mean effect size of a specific phenomenon and assess its variation across studies.^87^ Additionally, the contribution of different moderators (i.e., factors accounting for cross-study variation, such as type of NIRS machine used or age of participants) may be statistically evaluated and biases in the published literature like the “file drawer effect” (i.e., failure to publish null results) may be identified. Meta-analyses have been successfully conducted over behavioral data in developmental populations.^88^^,^^89^ To date, very few studies have carried out meta-analyses on developmental fNIRS data.^5^^,^^90^^,^^91^ These studies suggest that at least some of the published fNIRS results are robust to replication and have small-to-moderate effect sizes, which is typical of developmental studies conducted with other methods.^88^ Further studies will be needed in the future to systematically assess the replicability and effect sizes of key findings in the developmental fNIRS literature.

**Fig. 3:**
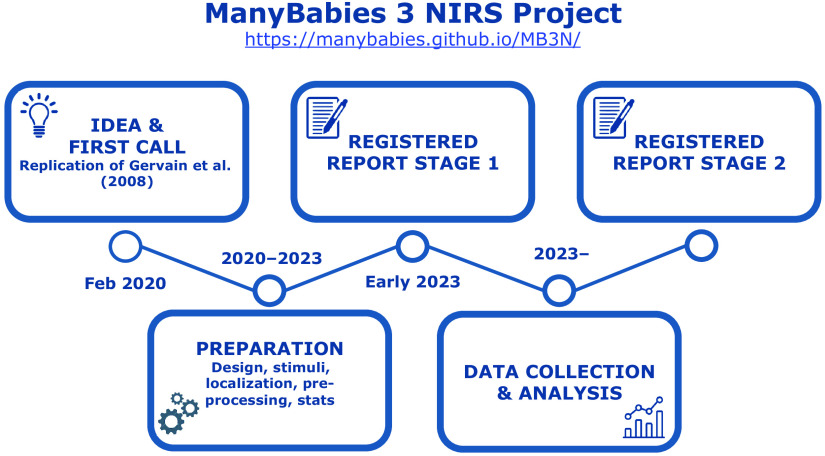
The timeline and progress of the ManyBabies 3 NIRS collaborative fNIRS replication project.

Collaborative, multilaboratory studies, whereby a given experimental protocol is carried out in a large number of different labs using methodologies as similar as possible is another approach to address the replicability crisis. Such studies, with their increased sample sizes, can provide higher statistical power and more robust conclusions than single studies. Such collaborative studies have successfully been conducted in many areas of general^83^ and developmental^92^ psychology. Currently, a collaborative, multilab replication project is ongoing aiming to address the cross-lab variability of young infants’ rule-learning ability in language as indexed by fNIRS. This ManyBabies 3 NIRS project^93^ (Fig. 3; https://manybabies.github.io/MB3N/) brings together 35 laboratories from 11 countries across 4 continents. The preparation and harmonization of research protocols is ongoing, and the resulting methods will be submitted as a registered report.

## 5 Discussion

The use of fNIRS to image the developing brain has grown considerably since its first applications nearly two and a half decades ago. Technological development and methodological innovations have allowed researchers to obtain increasingly high-quality data in the lab even from the very challenging populations of infants and toddlers. As we look into the future of developmental fNIRS, the next challenge is to take fNIRS studies out of the laboratories and into the wild. Moving away from the laboratory will allow the developmental fNIRS community to obtain a more accurate, less biased understanding of human neural development and to test participants while they are performing behaviors or are placed in settings that correspond more closely to real-life situations. Although these initiatives correspond to more general trends in psychology and neuroscience,^94^ fNIRS is particularly well-suited to achieve these goals. One consequence of moving research to more naturalistic and global settings is that researchers will have less control over the environmental variables, such as illumination, temperature, ambient noise, and distractors in the environment, that might influence infants’ behavior. As a consequence, variability in the data may increase (though see Ref. 95), making a focus on power and analytic techniques all the more important. Initiatives to conduct fNIRS in the wild go hand-in-hand with efforts to quantify variability, assess replicability, and increase sample sizes, again following more general tendencies in research with human participants.^86^ Indeed, standardization has been and will likely continue to be, a major goal in the field,^96^ and new data analysis techniques, statistical methods, and replication projects are emerging.

Overall, we see these advances and the new challenges in developmental fNIRS as an opportunity to analyze the brain as a multiscale system operating in a highly complex environment. Complementing more traditional localization-based approaches in single populations and age groups studied in a single lab with fNIRS as the only or main dependent measure, the field is moving toward the analysis of multimodal datasets where fNIRS data need to be combined with data from other imaging, physiological or behavioral measures. Furthermore, there is a drive to provide access to more standardized and openly communicated research procedures as well as to establish research hubs and networks that span multiple international sites. Developmental fNIRS is moving toward collecting data with numerous diverse populations giving us a more complex and complete picture of human neural development.

## 6 Conclusion

We see the advances and the challenges in developmental fNIRS discussed above as an opportunity to analyze the brain as a multiscale system operating in a highly complex environment. Complementing more traditional localization-based approaches in single populations and age groups studied in a single lab with fNIRS as the only or main dependent measure, the field is moving toward the analysis of multimodal datasets where fNIRS data need to be combined with data from other imaging, physiological or behavioral measures. Furthermore, there is a drive to provide access to more standardized and openly communicated research procedures as well as to establish research hubs and networks that span multiple international sites. Developmental fNIRS is moving toward collecting data with numerous diverse populations giving us a more complex and complete picture of human neural development.
